# Gambling Motives: Do They Explain Cognitive Distortions in Male Poker Gamblers?

**DOI:** 10.1007/s10899-017-9700-8

**Published:** 2017-06-17

**Authors:** Sasha Mathieu, Servane Barrault, Paul Brunault, Isabelle Varescon

**Affiliations:** 1 Laboratory of Psychopathology and Health Processes EA 4057, University Paris Descartes, Sorbonne Paris Cité, 71 Avenue Édouard Vaillant, 92100 Boulogne-Billancourt, France; 2 Laboratory of Ages of Life Psychology and Adaptation EA 2114, Psychology Department, University François Rabelais de Tours, 3 Rue des Tanneurs, 37041 Tours, France; 3 Equipe de Liaison et de Soins en Psychiatrie, Psychiatry Department, CHRU de Tours, 2 Boulevard Tonnellé, 37044 Tours, France; 4 Centre de Soins d’Accompagnement et de Prévention en Addictologie (CSAPA 37), CHRU de Tours, 37044 Tours Cedex, France

**Keywords:** Gambling, Gambling motives, Cognitive distortions, Problem gambling, Poker

## Abstract

Gambling behavior is partly the result of varied motivations leading individuals to participate in gambling activities. Specific motivational profiles are found in gamblers, and gambling motives are closely linked to the development of cognitive distortions. This cross-sectional study aimed to predict cognitive distortions from gambling motives in poker players. The population was recruited in online gambling forums. Participants reported gambling at least once a week. Data included sociodemographic characteristics, the South Oaks Gambling Screen, the Gambling Motives Questionnaire-Financial and the Gambling-Related Cognition Scale. This study was conducted on 259 male poker gamblers (aged 18–69 years, 14.3% probable pathological gamblers). Univariate analyses showed that cognitive distortions were independently predicted by overall gambling motives (34.8%) and problem gambling (22.4%) (*p* < .05). The multivariate model, including these two variables, explained 39.7% of cognitive distortions (*p* < .05). The results associated with the literature data highlight that cognitive distortions are a good discriminating factor of gambling problems, showing a close inter-relationship between gambling motives, cognitive distortions and the severity of gambling. These data are consistent with the following theoretical process model: gambling motives lead individuals to practice and repeat the gambling experience, which may lead them to develop cognitive distortions, which in turn favor problem gambling. This study opens up new research perspectives to understand better the mechanisms underlying gambling practice and has clinical implications in terms of prevention and treatment. For example, a coupled motivational and cognitive intervention focused on gambling motives/cognitive distortions could be beneficial for individuals with gambling problems.

## Introduction

Gambling is characterized as an activity whose outcome is based mainly or totally on chance, which involves an irreversible provision of money or an object of value beforehand. Different gambling types include games of luck (lottery, slot machines, scratch-cards and roulette) and games of skill (poker, blackjack, sports and horse betting). The outcome of games of luck is due only to chance, while games of skill depend on luck, strategy, experience and knowledge parameters. To date, few epidemiological data are available on European populations, which can be explained by the lack of research on the prevalence of gamblers in the general population. Different sets of practices can be identified depending on the intensity of gambling, defined by its frequency or the amount spent on games. In France and Northern Europe, 1–2% of problem or pathological gambling occurs in the general population. A similar prevalence was found in Canada and New Zealand (Costes et al. 2011). However, these estimations are much lower than those found in the United States and Australia (around 5%). Differences in terms of prevalence between countries are still widely discussed. Accessibility to gambling and the materials used to obtain these data are some explanations regularly put forward.

Among types of gambling, poker playing appears to have special features, particularly the involvement of both chance and strategy in the game’s outcome (Barrault et al. 2014), which could influence players’ perception of chance in the game (Barrault and Varescon 2013). Furthermore, poker players show specific gambling problems (Bjerg 2010; Barrault et al. 2014). Evidence from the literature thus suggests that excessive poker players may have a specific psychological profile.

While the practice of gambling can be seen as a personal choice, it can be influenced by factors favoring it or not (Burlacu et al. 2013). Cognitive distortions are one of the variables that influence involvement in gambling activity. Inherent to gambling situations, all gamblers, including non-problem gamblers and gamblers who have good numerical and objective probabilities of winning, are susceptible to developing cognitive distortions. These are therefore not related to a lack of knowledge or information regarding the game (Lambos and Delfabbro 2007). According to Sévigny and Ladouceur (2003), pathological gamblers will experience two cognitive states: one focused on taking into account rational and objective probabilities, and another primarily focused on the activity and the expected results. Thus, even individuals with a good knowledge of probabilities and principles could present cognitive distortions that would be activated in gambling situations (Barrault and Varescon 2013). These irrational beliefs coexist with and oppose the rational beliefs of the gamblers. This cognitive change can be explained by the concept of heuristic control. In fact, in a particular class of situations, such as gambling, individuals are more likely to overestimate the amount of their perceived control over results; particularly when they are strongly committed to the task and/or they have a strong desire for results (Clark 2014; Delfabbro et al. 2006).

The presence of such cognitive distortions in gamblers is widely documented in the literature (Clark 2014). Hence, a model representing the five major beliefs related to gambling may be clinically significant (Raylu and Oei 2004). *Gambling*-*related expectancies* is more about the perception of the expected impact on the game itself than the hope of happiness, pleasure, or other types of emotions with personal utility that can be derived from the game. *Illusion of control* corresponds to the perceived controllability over the results of a game, while *predictive control* is the perception of predicting the outcome of the game. The belief about the *inability to stop gambling* refers to the perception of being unable to resist an urge to gamble. Finally, *interpretative bias* is characterized by an attributional belief, that is to say promoting the successful continuation of gambling and allocating losses to external factors.

Furthermore, cognitive distortions increase with the severity of gambling: problem gamblers seem to have more than non-problem gamblers (Miller and Currie 2008). They tend to overestimate the role of skill in games and consider gambling a financially profitable activity. However, the desire to win money causes losses that can be fueled by cognitive distortions. Delfabbro et al. (2006) implicitly suggested that gambling motives could influence cognitive distortions, particularly the illusion of control.

Although motivations vary according to the type of population studied (age and gender) (Dowling 2013; Sundqvist et al. 2016), the type of game, although this is not studied here (Binde 2013; Lee et al. 2007), and the severity of gambling (Francis et al. 2014), some of them are found fairly consistently in the literature. The motivational factor most often raised is financial gain, but others have also been highlighted: for fun or amusement, against boredom (Lam 2007; Neighbors et al. 2002), to escape from routine (Loroz 2004), to socialize, for excitement (Lee et al. 2007), for the challenge (Chantal et al. 1994), to escape from stress (Lee et al. 2007) or to escape depressive affects (Stewart and Zack 2008). These motives seem to reflect the expectations of gamblers about, on the one hand, the positive aspects of the reward of gambling behavior and, on the other hand, its potential to reduce negative affects.

Several models have been proposed to explain what motivates and animates gamblers; those of Binde (2013) and Milosevic and Ledgerwood (2010), who identified enhancement, coping and social motivation. Enhancement motivation refers to the presence of sensation-seeking, impulsiveness, a sense of excitement felt for the game and coping. Gamblers wishing to regulate their emotional states would have a high level of this motivation whereas gamblers with a high level of coping would be characterized by a set of inappropriately used behaviors to escape their negative emotional state (Bonnaire et al. 2009; Vachon and Bagby 2009). Lastly, gamblers with a high level of social motivation play to establish a social affiliation (for fun with friends, for example) and would not present psychopathological disorders. Added to this is the financial motivation, already mentioned, which cannot be dissociated from this type of gambling (Dechant 2013).

To date, much empirical research has been carried out on cognitive distortions. However, no study has focused specifically on the link between these variables and gambling motives. Thus, our objectives were to assess gambling practice, gambling motives and cognitive distortions in terms of their presence and nature in gamblers, to compare them between the different types of gamblers, to determine the link between motivations and cognitions, and also to explain the nature of the relationship between gambling motivations and cognitive distortions. In view of these objectives, it was hypothesized that (1) problem gamblers would show a higher level of gambling motives and a higher level of cognitive distortions than non-problem gamblers; (2) there would be strong correlations between these two variables and with gambling severity; and (3) gambling motives would be a strong predictor of cognitive distortions.

## Methods

### Participants

Gambler participants were recruited between June 2016 and October 2016 through online gambling forums (Bet Clever, Club Poker). After receiving an authorization from the administrators, an announcement was posted on the “presentation” tab of the gambler community. Members interested in participating in the study had to click on the LimeSurvey^®^ link provided in order to access the study (information, consent forms and questionnaires). All participants were at least 18 years of age, fluent French speakers, and with a regular online or live gambling activity (at least once a week). This criterion of regularity has been used previously in the literature (Bonnaire et al. 2009; Barrault and Varescon 2012). Finally, 259 male poker gamblers responded to all the questionnaires including 24% of non-problem gamblers (assessed by the South Oaks Gambling Screen—SOGS score under 3), 61.7% of at risk-problem gamblers (SOGS score between 3 and 4) and 14.3% of problem gamblers (SOGS score equal to or higher than 5).

The Ethics Committee approved the study (IRB number: 20162200001072) and informed consent was obtained from all participants, who took part freely and voluntarily.

### Materials

#### Sociodemographic and Gaming Data

Age, gender, marital status, level of education, household composition, professional activity and social-professional category were assessed. Gaming data were obtained using a questionnaire especially designed for this study, including all types of gambling (live and online).

#### South Oaks Gambling Screen (SOGS)

The SOGS (Lesieur and Blume 1987) was validated in French by Lejoyeux (1999). This is a self-administered 20-item questionnaire that assesses lifetime and current (occurring in the last 12 months) gambling habits and problems. A score less than or equal to 2 corresponds to the absence of problem gambling, a score of 3 or 4 corresponds to risky or problematic gambling, while a score greater than or equal to 5 defines the individual as a probable pathological gambler. In our study, this questionnaire appears only relevant for the current period (in the last 12 months) so we chose to remove the lifetime assessment. The SOGS shows satisfactory reliability and validity in the general population and in problem gamblers (Stinchfield 2002). In the current sample, Cronbach’s alpha was .72, indicating good internal consistency.

#### Gambling Motives Questionnaire-Financial (GMQ-F)

The Gambling Motives Questionnaire (GMQ—Stewart and Zack 2008) is a 15-item questionnaire directly adapted from the Drinking Motives Questionnaire (Cooper et al. 1992) assessing three types of gambling motive: “enhancement”—to increase positive emotions (5 items), “coping”—to reduce or avoid negative emotions (5 items), and “social”—to increase social affiliation (5 items). Dechant (2013) then proposed adding 9 items to assess the financial motivation of gamblers, a non-negligible factor. The resulting GMQ-F tool was translated and validated in French (Devos et al. 2017). Items were rated on a 4-point Likert scale ranging from 1 (*never or almost never*) to 4 (*almost always or always*). This questionnaire has shown favorable psychometric properties on middle-aged adult gamblers (Dechant and Ellery 2011). In the present study, Cronbach’s alpha for the total scale (α = .80) and for each subscale was satisfactory: enhancement (α = .80), coping (α = .76), social (α = .65) and financial (α = .67).

#### Gambling Related Cognition Scale (GRCS)

The GRCS (Raylu and Oei 2004), validated in French (Grall-Bronnec et al. 2012), is a 23-item self-report that records common thoughts associated with problem gambling. This questionnaire uses a seven-point Likert scale ranging from 1 (*strongly disagree*) to 7 (*strongly agree*) that provides a total score (23–161). Items are grouped into five subscales: predictive control (6 items), illusion of control (4 items), interpretative bias (4 items), gambling expectancies (4 items), and inability to stop (5 items). The higher the score is, the greater the gambling-related cognitions are. In our study, the internal consistency for the total score (α = .84) and for each subscore was adequate: gambling expectancies (α = .62), illusion of control (α = .75), predictive control (α = .54), perceived inability to stop (α = .84), and interpretative bias (α = .57).

### Data Analyses

All data were analyzed using SPSS^®^, version 21, and were tested with a two-sided significance level of .05. Descriptive statistics for quantitative measures (mean, standard deviation) and for qualitative measures (percentage) were first calculated. To estimate the group effect regarding sociodemographic dimensions, and regarding the results of questionnaires, Chi squared tests were used for categorical measures and ANOVA for continuous ones (non-problem gamblers—NPGs vs. at risk-problem gamblers—RPGs vs. probable pathological gamblers—PPGs). Next, Spearman correlations were made between SOGS scores and the scores and subscores of the GMQ and GRCS. Then, one model of linear regression analysis was designed with the gambling motives and the problem gambling as the independent variables. Separate models were calculated for each scale (SOGS and GMQ) and subscale score. Finally, a multivariate linear regression analysis was designed with all the predictors with a significant effect revealed in the previous independent models.

## Results

### Sociodemographic Characteristics and Gambling

Two hundred and fifty-nine (259) male poker gamblers completed the online questionnaires. Sociodemographic data, SOGS scores and the characteristics of gambling practices are detailed in Table 1. For the entire sample, the average age was 33.9 years (SD = 9.3). Statistical analyses (ANOVA and Chi^2^) showed significant differences between NPGs (n = 62), RPGs (n = 160) and PPGs (n = 37) only for professional activity and socio-professional category in terms of sociodemographic variables (*p* < .05).

**Table 1:** Sociodemographic data and gambling practices

	NPG (n = 62)	RPG (n = 160)	PPG (n = 37)	Total (n = 259)	ANOVA	*p*
	Mean (SD)	Mean (SD)	Mean (SD)	Mean (SD)	F	
Age	37.7 (10.3)	32.5 (8.9)	33.8 (8.1)	33.9 (9.3)	7.329	.001*
SOGS	.0 (.0)	2.0 (1.1)	7.1 (2.8)	2.2 (2.5)	319.957	.000**
	N (%)	N (%)	N (%)	N (%)	χ^2^	*p*
Education					14.316	.074
No graduate certificate	0 (.0)	1 (.6)	0 (.0)	1 (.4)		
<High school degree	8 (12.3)	7 (4.3)	5 (12.8)	20 (7.5)		
High school degree	12 (18.5)	28 (17.8)	8 (25.6)	48 (19.1)		
Graduate study degree	39 (64.6)	123 (76.7)	24 (61.5)	186 (71.5)		
Others	3 (4.6)	1 (.6)	0 (.0)	4 (1.5)		
Family situation					8.575	.199
Single	25 (41.5)	79 (49.7)	15 (43.6)	119 (46.8)		
Married or in a couple	33 (52.3)	73 (45.4)	20 (51.3)	126 (47.9)		
Divorced	2 (3.1)	8 (4.9)	2 (5.1)	12 (4.5)		
Widowed	2 (3.1)	0 (.0)	0 (.0)	2 (.7)		
Professional activity					28.313	.013*
Full-time	37 (60.0)	83 (51.5)	26 (66.7)	146 (55.8)		
Part-time	1 (1.5)	8 (4.9)	0 (.0)	9 (3.4)		
Irregular	6 (9.2)	13 (8.0)	1 (5.1)	20 (7.9)		
Unemployed	8 (12.3)	11 (6.7)	6 (15.4)	25 (9.4)		
Student	3 (6.2)	26 (16.6)	3 (10.3)	32 (13.1)		
Disability/long-term sick leave	0 (.0)	2 (1.8)	0 (.0)	2 (1.1)		
Retired	4 (6.2)	1 (.6)	0 (.0)	5 (1.9)		
Other	3 (4.6)	16 (9.8)	1 (2.6)	20 (7.5)		
Socio-professional category					25.451	.030*
Agricultural worker	0 (.0)	1 (.6)	0 (.0)	1 (.4)		
Craftsman	4 (7.7)	13 (8.6)	1 (2.6)	18 (7.5)		
Executive	23 (38.5)	67 (41.1)	12 (30.8)	102 (39.0)		
Intermediate prof.	7 (10.8)	10 (6.7)	4 (12.8)	21 (8.6)		
Employee	16 (24.6)	20 (12.3)	7 (17.9)	43 (16.1)		
Workman	4 (6.2)	8 (4.9)	7 (17.9)	19 (7.1)		
Retired	1 (1.5)	0 (.0)	0 (.0)	1 (.4)		
Others without prof.	7 (10.8)	41 (25.8)	6 (17.9)	54 (21.0)		
Gamblers with children	26 (41.5)	50 (31.3)		15 (34.8)	2.792	.248

*NPG* Non-problem gamblers, *RPG* Risk-problem gamblers, *PPG* problem gamblers, *Others without prof.* includes students. ** *p* < .001; * *p* < .05

### Scale and Questionnaire Results

The results from each scale and the comparison between groups (ANOVA) are presented in Table 2. Table 3 shows the correlations between scales and subscales.

**Table 2:** SOGS, GMQ and GRCS scores and subscores

	NPG (n = 65)	RPG (n = 163)	PPG (n = 39)	Total (N = 267)	F	*p*
SOGS mean (SD)	.0 (.0)	2.0 (1.1)	7.1 (2.8)	2.2 (2.5)	319.957	.000**
GMQ mean (SD)	40.8 (7.0)	45.7 (7.9)	52.8 (11.0)	45.5 (8.9)	24.8	.000**
Enhancement	12.7 (3.4)	13.8 (3.5)	14.8 (3.6)	13.7 (3.5)	4.5	.012*
Coping	7.6 (2.1)	8.3 (2.8)	11.3 (3.5)	8.6 (3.0)	21.9	.000**
Social	5.5 (2.0)	5.6 (1.7)	6.3 (2.3)	5.7 (1.9)	2.4	.088
Financial	15.1 (4.0)	18.0 (4.0)	20.5 (5.9)	17.6 (4.6)	19.1	.000**
GRCS mean (SD)	54.2 (12.6)	64.1 (14.7)	79.3 (23.6)	63.9 (17.5)	29.0	.000**
Gambling-related expectancies	12.4 (3.7)	14.2 (4.4)	16.6 (5.5)	14.1 (4.6)	10.7	.000**
Illusion of control	5.1 (1.9)	5.2 (2.6)	7.9 (5.7)	5.6 (3.2)	12.0	.000**
Predictive control	14.3 (4.8)	16.0 (5.1)	17.4 (6.7)	15.8 (5.4)	4.1	.018*
Inability to stop gambling	8.7 (3.9)	12.5 (6.0)	20.9 (7.3)	12.8 (6.8)	52.8	.000**
Interpretative bias	13.7 (4.5)	16.1 (4.9)	16.6 (5.1)	15.6 (4.9)	6.3	.002*

*NPG* non-problem gamblers, *RPG* risk of problem gamblers, *PPG* problem gamblers, *SOGS* South Oaks Gambling Screen, *GMQ* Gambling Motives Questionnaire, *GRCS* Gambling-Related Cognitions Scale. ** *p* < .001; * *p* < .05

**Table 3:** Correlations between GMQ and GRCS total and subscales

	SOGS	GRCS	GRCS1	GRCS2	GRCS3	GRCS4	GRCS5
SOGS	1	.39**	.23**	.15*	.13*	.49**	.16*
GMQ	.39**	.56**	.51**	.24**	.31**	.46**	.25**
GMQ enhancement	.21**	.37**	.39**	.07	.19**	.31**	.14*
GMQ coping	.32**	.42**	.44**	.19**	.17**	.40**	.14*
GMQ social	.10	.29**	.24**	.22**	.15*	.25**	.11
GMQ financial	.34**	.40**	.32**	.20**	.29**	.28**	.26**

*SOGS* South Oaks Gambling Screen, *GRCS* Gambling-Related Cognitions Scale, *GRCS1* Gambling-related expectancies, *GRCS2* Illusion of control, *GRCS3* Predictive control, *GRCS4* Inability to stop gambling, *GRCS5* Interpretative bias, *GMQ* Gambling Motives Questionnaire. ** *p* < .001; * *p* < .05

#### Gambling Motives Questionnaire-Financial (GMQ-F)

The ANOVA results revealed that PPGs had significantly higher scores than the two other groups for the GMQ overall score (*p* = .001) and all subscores (*p* = .012 for coping and *p* = .001 for the others) except for “social” (*p* = .088, *p* > .05).

#### Gambling-Related Cognitions Scale (GRCS)

The ANOVA showed that PPGs displayed significantly higher GRCS scores than the two other groups (*p* = .001), for the general scale and for all subscales (*p* value ranging from .001 to .18).

In general, gambling motives (except social motivation) and cognitive distortions increased with the severity of the gambling practice (*p* < .05). Moreover, significant correlations between problem gambling, cognitive distortions and gambling motives were observed (*p* < .05). Gambling motives (overall GMQ-F) appeared to be significantly and strongly correlated with a specific dimension of cognitive distortions (i.e. gambling-related expectancies) (r = .51, *p* < .05).

### Multiple Linear Regression

A three-step regression was conducted. First, a simple linear regression model was carried out to determine whether the components of gambling motives (independent variable) could predict problem gambling (SOGS score). It showed that overall motivations explained 22.0% of probable pathological gambling in our sample (Adjusted R^2^ = .217; t = 8.521; *p* = .001). In gambling motives, coping appeared to be the main significant predictor (17.7%; R^2^ = .174; t = 7.444; *p* = .001) of probable pathological gambling, followed closely by financial motivation (17.2%; R^2^ = .169; t = 7.304; *p* = .001). The second step included, one by one, sociodemographic variables, gambling motives (scale and subscales) and the SOGS score as predictors of cognitive distortions. The results showed that no sociodemographic variables significantly predicted cognitive distortions (*p* > .05). However, on their own, gambling motives (overall GMQ-F) explained 34.8% of cognitive distortions present in our sample (Adjusted R^2^ = .345; t = 11.712; *p* = .001), whereas problem gambling (overall SOGS) explained 22.4% of these cognitive distortions (Adjusted R^2^ = .221; t = 8.609; *p* = .001). Although this model was statistically significant, two dimensions of gambling motives, coping and financial, constituted significant predictors (22.3 and 21.4%, respectively) of cognitive distortions (Adjusted R^2^ = .220; t = 8.588 for coping, and Adjusted R^2^ = .211; t = 8.373 for financial; *p* = .001 for both).

Finally, a multivariate analysis was conducted (see Table 4). All the significant predictors listed below were entered simultaneously with cognitive distortions as the dependent variable. This multivariate model accounted for 39.7% of the variance of cognitive distortions (Adjusted R^2^ = .393; *p* < .05). However, only three significant predictors were highlighted: overall gambling motives and problem gambling were positively related to cognitive distortions (respectively, t = 8.586 and t = 4.580; *p* = .001), while “enhancement” motivation was related negatively to cognitive distortions (t = −2.400; *p* < .05).

**Table 4:** Multivariate analyses

GRCS (dependent variable)	*R* ^*2*^	Adjusted *R* ^2^	Bêta	t	*p*
*Model with subscores (predictors)*					
GMQ	.397	.393	.472	8.586	.000*
Enhancement			−.179	−2.400	.017*
Coping			.073	.970	.333
Social			−.073	−1.499	.135
Financial			.121	1.784	.076
SOGS			.252	4.580	.000*

*SOGS* South Oaks Gambling Screen, *GRCS* Gambling-Related Cognitions Scale, *GMQ* Gambling Motives Questionnaire. * *p* < .05

## Discussion

This research is the first to assess gambling motives and cognitive distortions jointly in poke players. One of the purposes of this study was to investigate whether gambling motives explain cognitive distortions.

The main results show that gambling motives are a strong predictor of cognitive distortions. This close linkage enables us to hypothesize that gamblers with more motives to gamble (both in quality and in quantity) are more likely to develop and maintain cognitive distortions during gambling, such as the failure to appreciate the independence of turns (GRCS predictive control) and expectancies that gambling will be exciting and/or relieve negative affect (GRCS gambling related-expectancies) for example. Cognitive distortions are mainly explained by financial and coping motivation. Greed and the tendency to escape from daily difficulties may explain the presence of more or fewer cognitive distortions. Indeed, the desire to succeed and win money may be overestimated by gamblers while creating financial losses. Moreover, winning money probably enables some gamblers to escape their routine and their difficulties, which may reinforce cognitive distortions. In addition, the game’s outcome modulates the gambler’s mood: more depressive affects are observed after a loss, and more euphoria after a win.

The link between gambling motives and gambling severity has already been studied in the literature (Francis et al. 2014). Our results confirm these results and show that gambling motives are significantly correlated to the degree of gambling severity. Moreover, the differences between the three groups of gamblers (NPGs, RPGs and PPGs) are statistically significant for all dimensions of the GMQ-F scale, except for “social” motivation. Earlier research showed that PPGs gamble more to escape problems compared to recreational gamblers (Burlacu et al. 2013; Clarke 2008; Platz and Millar 2001), which is supported in this study. Respondents with a gambling problem have significantly higher scores than RPGs and NPGs: first, on coping, secondly, on financial motivation and then on enhancement. The content of the coping subscale might mediate the negative affects found in pathological gamblers (Connor-Smith and Flachsbart 2007). Furthermore, our study highlights the nature of the relationship between these two variables: gambling motives explain 22.0% of problem gambling. Individual differences, particularly in terms of motives, may be a vulnerability factor of problem gambling behavior.

Multivariate analyses reveal that gambling severity associated with gambling motives accounts for 39.7% of cognitive distortions, with the enhancement motive negatively related to cognitive distortions. We can hypothesize that the acquisition of a set of knowledge and know-how, in order to progress, opposes the potential installation of cognitive distortions. For example, gamblers motivated by improvement are well informed about the game (probabilities, gambling techniques, etc.). Nevertheless, our sample is mainly composed of male gamblers, who are more oriented towards, on the one hand, games of skill (poker and sports betting), and on the other hand, enhancement (Gandolfo and Debonis 2014). The enhancement motives may reflect a desire to increase self-esteem. However, changes in self-perception are directly linked to major trends in success and failure, also related to the acquired skills.

Nevertheless, the literature also reports the link between the severity of gambling and cognitive distortions, putting forward cognitive distortions as a risk factor and not the other way round (Barrault and Varescon 2013; Cunningham et al. 2014; Navas et al. 2016; Romo et al. 2016). In our study, all GRCS measures were higher in the PG group than in the other two groups (NPGs and RPGs). The inability to stop gambling appears as the main reported cognitive distortion. However, this cognitive distortion component is one of the criteria characterizing pathological gambling. This result highlights a certain awareness in problem gamblers of their difficulties in controlling their gambling behavior. Unlike the results found previously, in pathological gamblers (Michalczuk et al. 2011; Navas et al. 2016) and pathological gambling poker players (Barrault and Varescon 2013), the illusion of control does not appear to be an important belief. However, it is possible that gamblers subjectively experience the illusion of control, without it concretely impacting their way of gambling. Gamblers could also have underestimated their answers as they were not in gambling conditions when filling out the questionnaires. This is consistent with the results found by Dannewitz and Weatherly (2007). Moreover, several factors may influence cognitive distortions, including the degree of involvement of the gambler and some features of the game (Ladouceur and Sévigny 2005). The literature highlights, on one hand, a quantitative difference in cognitive distortions depending on game frequency and intensity (the level of irrational beliefs increases with the intensity of gambling practice) and on the other hand, a qualitative difference (the role of skill in games and the perception of the game as financially profitable are more present in pathological gamblers) (Delfabbro et al. 2006; Källmen et al. 2008; Miller and Currie 2008; Moodie 2008; Raylu and Oei 2004). Different groupings of preferred gambling type, such as games of skill and games of luck, could be another way to examine cognitive distortions.

To our knowledge, few studies have focused on gambling motives. This is why our study aimed to provide some additional information by identifying and comparing gambling motivations based on gambling practices. However, there are several limitations to the interpretation and generalization of the results. First, participants are only male poker gamblers, who are not representative of the gambler population due to their specific profile. In order to limit this bias, studies need to be carried out on groups of gamblers created according to the type of game practiced. Moreover, we cannot generalize the results to women gamblers while a gender effect has been demonstrated in some previous studies. In fact, in both pathological and non-pathological gamblers, women appear to have less intense cognitive distortions than men (Dannewitz and Weatherly 2007; Moodie 2008; Raylu and Oei 2004). Nevertheless, we tried to constitute a sample as representative as possible by recruiting our participants in ecological settings (Internet forums). Secondly, we used the SOGS (Lesieur and Blume 1987) to screen for gambling problems: in our sample, we observed 14.3% of probable pathological gamblers, which is similar to the results found by Barrault and Varescon (2013) using the SOGS, and by Wood et al. (2007) using the DMS-IV-TR criteria (APA 2003). Although the SOGS is known to foster false positives in a general population (Stinchfield 2002), it is still the most used screening tool in research. Another limitation of this study is the cross-sectional design. Indeed, it seems to make changes in motivations and/or in involvement over time and in terms of gambling practice. A longitudinal or qualitative study would allow access to these evolutionary elements. Moreover, for practical reasons, our results are based on the prevalence during the current period (the last year). Finally, we were unable to compare online and live gamblers because the majority of our participants reported having both types of practices. Another approach could be to compare, as far as possible in terms of feasibility, exclusive online gamblers and live gamblers. Despite these limitations, the present study offers interesting results and research perspectives. The relationship between gambling problems in all gamblers, gambling motives and cognitive distortions merits further study.

## Conclusions and Implications

In conclusion, gambling motives differ between gamblers depending on the intensity and severity of the gambling practice. The level of risky gambling is associated with motivations and should be considered during tests or assessment. Moreover, the presence of cognitive distortions is impacted by the presence of gambling motives. A longitudinal study would show the evolution of motivations and cognitive distortions (in terms of numbers and importance) in gamblers engaging in regular gambling activity.

The differences in psychological functioning between non-problem gamblers, at risk-gamblers and those with a probable problem demonstrate the need for further research to understand better the factors involved in gambler behavior, with the objective of prevention and care for gamblers. With these results, we can hypothesize that a systematic assessment of gambling motives of gamblers would enable us to target the type of intervention required. Moreover, due to the possible presence of varying levels of cognitive distortions, a motivational and cognitive intervention (such as cognitive remediation) would act on the two components of gambling, directly linked to gambling problems.
